# Disease Activity and Conversion into Multiple Sclerosis after Optic Neuritis Is Treated with Erythropoietin

**DOI:** 10.3390/ijms17101666

**Published:** 2016-09-30

**Authors:** Kurt-Wolfram Sühs, Panagiotis Papanagiotou, Katharina Hein, Refik Pul, Kerstin Scholz, Christoph Heesen, Ricarda Diem

**Affiliations:** 1 Department of Neurology, Medical School Hannover, Carl-Neuberg Str. 1, 30625 Hannover, Germany; pul.refik@mh-hannover.de; 2 Department of Neuroradiology, Hospital Bremen Mitte, St. Jürgen Str. 1, 28177 Bremen, Germany; panagiotis.papanagiotou@klinikum-bremen-mitte.de; 3 Department of Neurology, Georg-August University, Robert-Koch-Str. 40, 37075 Göttingen, Germany; k.hein@uni-goettingen.de; 4 Department of Radiology, Lüneburg Hospital, Bögelstr. 1, 21339 Lüneburg, Germany; ke.scholz@asklepios.com; 5 Department of Neurology, University Hospital Hamburg-Eppendorf, Martinistr. 52, 20246 Hamburg, Germany; heesen@uke.de; 6 Department of Neurooncology, University Clinic Heidelberg, Im Neuenheimer Feld 400, 69120 Heidelberg, Germany; ricarda.diem@med.uni-heidelberg.de

**Keywords:** multiple sclerosis, MRI, optic nerve, clinical trial

## Abstract

Changes in cerebral lesion load by magnetic resonance imaging (MRI) in patients from a double-blind, placebo-controlled, phase II study on erythropoietin in clinically isolated optic neuritis (ClinicalTrials.gov, NCT00355095) were analyzed. Therefore, patients with acute optic neuritis were assigned to receive either 33,000 IU of recombinant human erythropoietin (IV) daily for three days, or a placebo, as an add-on to methylprednisolone. Of 35 patients, we investigated changes in cerebral lesion load in MRIs obtained at baseline and at weeks 4, 8, and 16. In 5 of the 35 patients, we found conversion into multiple sclerosis (MS) based on MRI progression only. These five patients had received the placebo. Another five patients showed MRI progression together with relapses. Three of these patients had received erythropoietin, and two the placebo. Yet, analyzing the change in absolute numbers of periventricular, juxtacortical, and infratentorial lesions including gadolinium-enhancing lesions, there were no significant differences between the groups. Although effective in terms of retinal nerve fiber layer protection, erythropoietin treatment of acute isolated optic neuritis did not influence further evolution of MRI lesions in the brain when comparing absolute numbers. However, early conversion from clinically isolated syndrome to MS assessed by MRI activity seemed to occur more frequently in the placebo-treated group.

## 1. Introduction

In a phase II pilot trial in isolated optic neuritis, we have shown that treatment with erythropoietin in addition to methylprednisolone pulse therapy exerts protective effects on the retinal nerve fibre layer (RNFL) and the optic nerve (ON) [1]. The RNFL contains the axons of retinal ganglion cells (RGCs) and degenerates after an episode of optic neuritis [2,3]. RNFL degeneration has been acknowledged to be an indicator of neurodegeneration in multiple sclerosis (MS) [4]. The mechanisms by which erythropoietin protects the RNFL and the ON are not fully clear but might involve neurotrophin-like effects on RGCs [5], or optimization of central nervous system CNS oxygen supply [6].

In this study, we used clinical and magnetic resonance imaging (MRI) data obtained during the erythropoietin treatment trial [1] in order to investigate (i) the influence of erythropoietin on conversion of clinically isolated optic neuritis into MS and (ii) its effects on cerebral lesion load and the numbers of gadolinium-enhancing lesions during the 16 weeks following treatment.

## 2. Results

### 2.1. Baseline Status

**Figure 1:**
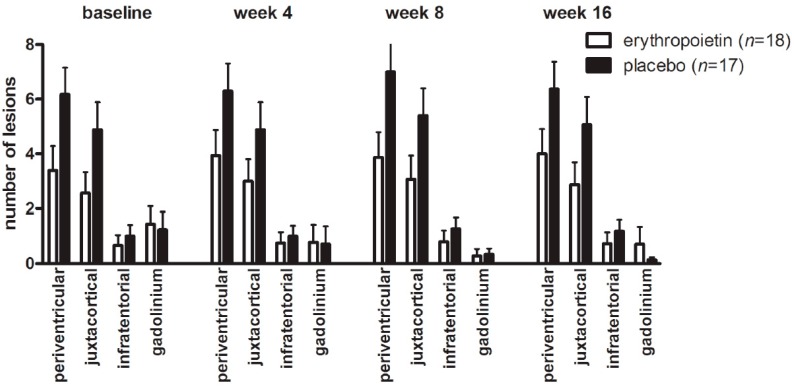
Cranial MRI lesion load. Mean T2 lesion load aligned by lesion site and gandolinium enhancing lesions at different time points. Empty bars = erythropoietin; black bars= placebo; error bars = SEM.

Complete MRI data (baseline and follow-ups at weeks 4, 8 and 16) were obtained in 35 patients (erythropoietin/placebo 18/17). At baseline, 10 patients (29%) had more than 10 subclinical T2 lesions, unequally distributed amongst the groups with 3 patients in the erythropoietin and 7 patients in the placebo group. At baseline, gadolinium-enhancing lesions outside the ON were found in almost one third of all patients (*n* = 10) distributed almost equally amongst the erythropoietin and the placebo group (erythropoietin/placebo: 6/4). In both groups, most lesions were located around the ventricles followed by juxtacortical and infratentorial lesions (Figure 1). Five patients (14%) did not show any brain lesions at baseline (erythropoietin/placebo: 3/2). Gadolinium enhancement within the ON was detected in 32 patients (91%) with only two patients in the erythropoietin and one in the placebo group, showing no active lesions within the ON.

### 2.2. Changes in Cerebral Lesion Load

MRI progression, defined as any new lesion within 16 weeks of follow up, was found in 10 patients (erythropoietin/placebo: 4/6) with 8 patients (erythropoietin/placebo: 4/4) showing new gadolinium-positive lesions. In five of these patients (erythropoietin/placebo: 3/2), gadolinium-enhancing lesions were found as early as week 4. In the majority of patients (7 out of 10; erythropoietin/placebo: 3/4), new lesions were observed until week 8. Patients who did not show T2-lesions at baseline remained stable during the course of the study. In patients with MRI progression, there was no significant difference in the numbers of T2-lesions between erythropoietin-treated (*n* = 4) and placebo-treated patients (*n* = 6). In both groups, an increase in lesion load was observed over the 16-week duration of the study (erythropoietin periventricular + 0.6111 ± 1.341 vs. placebo periventricular + 0.1985 ± 1.424, Figure 1). Additionally, we detected an increase in the number of juxtacortical lesions over the 16 weeks in both groups (erythropoietin juxtacortical + 0.3111 ± 1.182 vs. placebo juxtacortical + 0.1801 ± 1.457, Figure 1) and also a slight increase in infratentorial lesions (baseline erythropoietin + 0.0667 ± 0.585 vs. placebo + 0.1875 ± 0.5949). In general, the presence of infratentorial lesions correlated to a higher total lesion load (r = 0.587, *p* < 0.01). A similar correlation was found for periventricular (r = 0.666, *p* < 0.01) and juxtacortical lesions (r = 0.690, *p* < 0.01).

### 2.3. Conversion into MS

Based on the revised McDonald criteria [7], MS was diagnosed in 10 patients at baseline. During the duration of the study, MS diagnoses were made in another ten (29%) patients. In five of these patients (erythropoietin/placebo: 3/2), the diagnosis was based on a second relapse. In these patients, new MRI lesions were also observed. In the other five patients (erythropoietin/placebo: 0/5), diagnoses were made based on MRI progression according to the revised McDonald criteria.

The Expanded Disability Status Scale (EDSS) score did not differ between the treatment groups at week 16 (erythropoietin 0.9500 ± 0.2862 vs. placebo 1.360 ± 0.2805, *p* = 0.3150; mean ± SEM) and did neither correlate with lesion load at baseline nor at week 16.

## 3. Discussion

In our study, MRI was performed repeatedly at early time points and offers insights into early clinically isolated syndrome (CIS) in a homogenous patient group. In the literature, 50%–70% of CIS patients present with asymptomatic MRI lesions [8,9,10]. We observed T2 white matter lesions in 86% of our patients. Almost one third (29%) presented with gadolinium-enhancing lesions outside the ON. The high number of patients with asymptomatic T2- and gadolinium-enhancing lesions is probably due to the inclusion of patients with severe optic neuritis only.

Within the observational period of 16 weeks, in 10 patients (29%), diagnoses of MS were made by using the revised McDonald criteria [7]. Compared with long-term studies with larger MRI examination intervals, conversion rates of 26% after 1 year and of 65%–80% 7–20 years after CIS were observed [11,12,13]; following isolated optic neuritis, conversion rates of 30%–50% within 5 to 15 years were shown [14,15]. Using early time-points for MRI, we found a high rate of patients who had converted into MS already within the 16 weeks. The fact that most of the new MRI lesions occurred between week 4 and 8 implies that the early use of follow-up MRI may already be useful to make the diagnosis of MS in patients with severe ON. Only one other study presented data of monthly MRI after CIS, showing a conversion rate of 39% within 18 months; however, in this study, the time from first clinical event to first MRI was more variable (mean of 4.4 months) [12].

In our study, 7 out of 10 patients who converted into MS had received the placebo, whilst the other 3 received erythropoietin. In order to further investigate our observations, which are limited by the comparably small group size, we have currently started a large phase III trial on erythropoietin in optic neuritis (ClinicalTrials.gov, NCT01962571). However, in our pilot trial, we did not find any significant differences when comparing cerebral lesions over time between the two patient groups. This is in line with results from testing erythropoietin in an animal model of optic neuritis where it did not exert any anti-inflammatory or immunosuppressive effects [5]. Erythropoietin in our phase II pilot trial was applied over only 3 days, and the results confirm our hypothesis that erythropoietin exerts neuroprotective properties mainly in an acute setting.

## 4. Materials and Methods

The trial protocol has been published [1] and only pertinent aspects are summarized here. Patients aged between 18–50 years with a first episode of optic neuritis, a decreased visual acuity to ≤0.5 (decimal system), and onset of symptoms within the last 10 days have been included in this double-blind, placebo-controlled, parallel group study. The study was approved and monitored by the Ethics Committee of the University of Göttingen (as the central Ethics Committee) and the German Federal Institute for Drugs and Medical Devices, Bonn. The study is registered with ClinicalTrials.gov, NCT00355095. All participants gave written informed consent. Patients were randomly assigned (1:1) to erythropoietin or placebo stratified according to center (University Clinic Homburg/Saar, Göttingen, or Hamburg; Germany). Recombinant human erythropoietin (33,000 IE Epoetin-alpha, ErypoFS^®^, Janssen Cilag) as study medication, or placebo (0.9% NaC), was applied as an IV injection once daily after the application of methylprednisolone (1000 mg iv per day; Urbason^®^ solubile forte, Aventis Pharma) on three consecutive days. Study medication was given after the application of methylprednisolone (1000 mg IV per day; Urbason^®^ solubile forte, Aventis Pharma). MRI was done at baseline (prior to treatment) and at weeks 4, 8, and 16 to assess changes in the numbers of periventricular, juxtacortical, and infratentorial T2-lesions and gadolinium-enhancing lesions. MRI was performed on 1.5T machines (Siemens Magnetom Sonata/Siemens Avanto, Erlangen, Germany; centres Homburg/Saar and Hamburg) or on a 3T machine (Siemens TimTrio; centre Göttingen, Germany). In each patient, the complete set of examinations was performed on the same scanner. The following images were obtained: 3-mm slices from transversal PD/T2-weighted turbo spin echo (TSE), from sagittal T2-weighted TSE, and from a sagittal T1-weighted 3D-sequence with gadolinium-diethylene triamine pentaacetic acid (DTPA). As orbital sequences, we used 2-mm slices from fat-suppressed coronal T2-weighted spin echo/turbo spin echo, from fat-suppressed transversal T2-weighted TSE, and from fat-suppressed coronal and transversal T1-weighted spin echo with gadolinium-DTPA. MRIs were analyzed centrally by a reader blinded for treatment allocation and double-checked for accuracy by another reader blinded for treatment and the results of the first reader. Inter-reader variability was low (correlation coefficient of 0.98).
